# Why Current Statistics of Complementary Alternative Medicine Clinical Trials is Invalid

**DOI:** 10.3390/jcm7060138

**Published:** 2018-06-07

**Authors:** Maurizio Pandolfi, Giulia Carreras

**Affiliations:** 1 Former Professor of Ophthalmology, University of Lund, SE-22100 Lund, Sweden; mauri.pandolfi@gmail.com; 2 Oncologic Network, Prevention and Research Institute, 50141 Florence, Italy

**Keywords:** complementary alternative medicine (CAM), *p*-value, Bayesian statistics, scientific plausibility

## Abstract

It is not sufficiently known that frequentist statistics cannot provide direct information on the probability that the research hypothesis tested is correct. The error resulting from this misunderstanding is compounded when the hypotheses under scrutiny have precarious scientific bases, which, generally, those of complementary alternative medicine (CAM) are. In such cases, it is mandatory to use inferential statistics, considering the prior probability that the hypothesis tested is true, such as the Bayesian statistics. The authors show that, under such circumstances, no real statistical significance can be achieved in CAM clinical trials. In this respect, CAM trials involving human material are also hardly defensible from an ethical viewpoint.

## 1. Introduction

“Suppose some dark night a policeman walks down a street apparently deserted. Suddenly, he hears a burglar alarm, looks across the street, and sees a jewelry store with a broken window. Then a man wearing a mask comes crawling out through the broken window carrying a bag…”

The treatise, “Probability Theory”. The Logic of Science” [1] by the physicist E.T. Jaynes, opens with this fictitious situation. Obviously, the policemen draw the preliminary conclusion that he is witnessing a burglary and intervenes to arrest the suspect. However, as it turns out, the situation is completely different: actually, the suspect is the owner of the jewelry store and he was coming home from a masquerade party; just as he walked by his store a passing truck threw a stone through the window and he was only protecting his own property.

We are, thus, facing two hypotheses: one very likely and the other very unlikely, although still possible. We, therefore, understand the following decision of the policemen to select the first one and act accordingly.

Ordinary clinical research deals with science-based, credible hypotheses. Thus, it can be justified to test a new anticoagulant for the prevention of thrombosis when we know in advance the biochemical properties of the drug and its mode of interfering with the coagulation cascade. Likewise, a new adrenergic beta-blocker can be suitably examined for glaucoma treatment, since this class of drugs is known to lower the intraocular pressure by decreasing the production of aqueous humor. In both examples, any favourable effect observed in the treated group can be reasonably ascribed to the specific action of the substance employed.

Until recently, this has been a general rule in clinical research and standard statistics, specifically, the *t* test, has been commonly used to evaluate the results obtained. Remarkably, it is, generally, ignored that inferential statistics expressing results as *p*-values cannot be used as direct support to a research hypothesis. As the method is devised, the *p*-value is not the probability that the research hypothesis is correct, but the probability to obtain the same or a more evident result when repeating the identical experiment—provided that there is no difference between the terms compared (null hypothesis or H0). Thus, a “significant” *p*-value as 0.01 does not represent the (in this case, low) probability that the difference observed between groups results from chance/is a product of background noise. Such interpretation, also known as the “*p*-value fallacy”, implies the error of “transposition of conditioning”: what we get is not the probability of the hypothesis given the data (i.e., what we actually wish to know) or Pr (H0|data), but the probability of the data given the null hypothesis or Pr (data|H0), where we indicated the probability with “Pr” and the conditioning with “|” [2,3]. Unfortunately, in clinical research the former is just the way the *p*-value is still understood and a “significant” *p*-value (0.05 or less) is routinely taken as evidence of the correctness of the hypothesis tested.

Statisticians are very strict on this point and, recently, the rationale behind an event of historical importance as the final validation of Higgs boson, announced by the European Organization for Nuclear Research (CERN), has been criticized because of the statistics used [4].

Fortunately, in the case of common sound medical research, this misunderstanding has not led to untoward consequences because the hypotheses tested have been biologically plausible and, therefore, correct in most cases. This favourable situation does not recur in the case of complementary alternative medicine (CAM) whose hypotheses (usually the specific efficacy of the treatment) are unlikely to be correct because they are biologically implausible. As Gorski and Novella noted [5], the biological plausibility of an intervention does not necessarily imply the knowledge of the exact mechanism of the action, but that, “the mechanism should not be so scientifically implausible as to be reasonably considered impossible”. This applies to all CAMs except phytotherapy for which the main objection regards the crudeness of its preparations. In these cases, the probability that a hypothesis of a specific efficacy is correct is even smaller than that of the masked jeweler protecting his own property, which, although infinitesimal, is not unreasonable. Under such circumstances, a *p* < 0.05, or even 0.01, cannot be used even as suggestive support of the hypothesis of efficacy, since it is far more likely that any “significant” outcome is the consequence of other causes, such as bias or a placebo effect. 

In these cases, a proper statistical evaluation should consider the plausibility of the hypothesis tested or it is a priori probability. This goal can be achieved with Bayesian statistics, which permits prior beliefs to be updated in the evidence of new data, getting a posteriori probabilities of the hypothesis tested. In this paper, we plan to illustrate this method by providing examples on how it can be applied to most commonly used forms of non-conventional medicine.

## 2. Methods

The theorem of Bayes can help to translate the *p*-value into the posterior probability and ascertain what is the direct support of the *p*-value for the hypothesis tested. For this purpose, we commonly use a variant of the Bayes’ equation expressed in odds form, as is shown:

Posterior odds of H0 = Prior odds of H0 × Bayes factor

where Bayes factor (Bf) is the ratio between the two likelihoods, Pr (data|H0) and Pr (data|Ha) i.e., the probability of the data, given the null hypothesis, H0, divided by the probability of the data, given the alternative hypothesis, Ha. In essence, Bf is a quotient indicating how far apart are the odds we put on H0 before initiating the investigation (prior odds) from the odds after seeing the data or posterior odds [6]. As the quotient is formulated, the smaller the Bf, the smaller the support for H0. The Bf has a continuous scale and it is useful to summarize the Bf in terms of discrete categories of posterior probabilities of the null hypothesis. Jeffreys [7] provided a classification scheme for Bf values that associates Bf ranges to the strength of evidence for H0 (Table 1). 

The “minimum Bf” is the smallest amount of evidence that can be claimed for H0 and does not involve a specific prior probability distribution. Rather, it is a global minimum over all prior distributions. When statistical tests are based on a Gaussian approximation, the minimum Bf is calculated from the same information that goes into the *p*-value as exp (−0.5 z^2^), where “exp” is the base of natural logarithms and “z” is the deviation in standard errors from the null effect [6].

The minimum Bf formula can also be used if the most common *t* test is performed by substituting *t* for z [8]. Rouder provided a useful web applet to compute the Bayes factor for the *t* distribution [9]. Moreover, a simple manual of epidemiology [10] contains a table reporting the relations between *p*-values, minimum Bf, and posterior probabilities of the null hypothesis assuming a “neutral” prior odds of H0 of 1:1 and a “moderately skeptical” prior odds of 9:1. The table shows how relevant the general reduction in the inferential support provided by the Bayesian procedure is, in comparison to that given by the *p*-value. For example, assuming prior odds for H0 = 1:1, a *p*-value = 0.05, i.e., a current measure of significance, corresponds to a non-significant (0.13) support of the null hypothesis. The table also shows how inopportune it is to interpret *p*-values at face value, especially when they are calculated from results obtained testing hypotheses with low prior probability. Thus, a “significant” *p*-value = 0.05 corresponds to a posterior probability of the null hypothesis = 0.57 (slightly in favour of it) if we test a dubious research hypothesis to whose H0, being “moderately skeptical”, and we assign prior odds of 9:1. Even for a *p*-value as low as 0.001, the posterior probability of the null hypothesis is just significant (0.043) if the assumed prior odds for H0 is 9:1.

In this paper, we re-analysed, according to the Bayesian theory, the frequentist results expressed in terms of *p*-value for three studies of CAM and other fields [11,12,13,14].

## 3. Results

A randomized controlled trial on moxibustion in obstetrics, i.e., stimulation with hot mugwort of a foot acupuncture point aimed to correct a breech presentation, found a significant (*p*-value 0.01) lower proportion of cephalic version in the control group in comparison to the treated women [11]. The *p*-value = 0.01 would result in a posterior probability of only 0.26, the prior odds assumed for such an implausible intervention being quite low (9:1). 

The situation regarding the use of acupuncture in pain is only seemingly different. Here, an effect is possible, since the intervention may have a plausible mechanism of action (secretion of endogenous opioids). Thus, having a “neutral” attitude and assuming prior odds for H0 = 1:1, *p*-values of 0.001 (as reported in a study on tension headache [12]) would give a modestly significant (0.043) posterior probability. However, such direct statistical evidence would still not support the unlikely hypothesis tested (beneficial effect following readjustment of the balance of imaginary vital fluids), but rather the much more credible assumption of an unspecific placebo effect. As Ernst observed, acupuncture, being exotic, invasive, slightly painful, involving touch, and direct contact with a therapist, carries most of the features capable of eliciting a placebo effect [13]. 

Similar improper use of statistics may occur in other fields, with one example being a recent investigation aimed to verify the existence of precognition, i.e., the alleged psychic ability to see events in the future [14]. The author, the psychologist DJ Bem, conducted nine separate studies to see if future events, when known in advance, may retroactively affect people’s response and obtained statistical significance in eight experiments out of nine at the 0.05 significance level. Bem presented the result as evidence that many humans can directly perceive the future and not just predict it based on the past. The paper received various forms of criticism, with the main one being of the same kind that was addressed to CAM’s clinical trials, i.e., that ordinary inferential statistics cannot be applied to hypotheses having an extremely low scientific plausibility. Wagenmakers et al. [15] re-evaluated the data using a Bayesian *t*-test, which considers the prior probability that the hypothesis is correct. By computing the Bf based on the t value and the degrees of freedom for all nine of Bem’s studies, they found that, out of the nine experiments, only one yielded substantial evidence for Ha, with a Bf of 0.17, whereas three yielded substantial evidence for H0, with Bf of 3.14, 3.49, and 7.61, respectively (Table 1). 

## 4. Discussion and Conclusions

Medical hypotheses, commonly evaluated in CAM studies, are unlikely to be correct because they are biologically implausible. Let us consider acupuncture, which among all CAMs seems to enjoy higher consideration. According to its “rationale” inserting a needle in given points of the skin redresses a disturbed balance of the vital body fluids, Yin and Yang, thereby, restoring health. Why? Because so stated ancient Chinese medical texts, like the Yellow Emperor’s Classic of Internal Medicine. As for the mechanism? “It improves the flow of Chi adjusting the balance between its constituents Ying with the Yang” is the standard answer. Chi or Qi, according to the “millenary wisdom of China”, corresponds to the vital fluid, or “élan vital”, of some Western philosophy or to the “prana” of traditional Indian medicine, Ayurveda, or to a “spiritual energy” as postulated by Reiki, or by other forms of CAM. But, more precisely, in which way should this adjustment of vital fluid take place? No further explanation is provided, but never mind, the “ancient Chinese wisdom” knows. Incidentally, this explanation should be sufficient to also justify the use of acupuncture in in vitro fertilization, an indication not foreseen in the Yellow Emperors Book.

Now, which kind of plausibility may have a medical treatment based on such tenuous grounds and conceived in prescientific times when most elementary functions of the body were unknown or totally misunderstood?

When testing acupuncture, and other forms of CAM, more meaningful statistical significance would be obtained if the hypothesis on trial was not, as is customary in these studies, the specific action of the intervention, but its capacity to elicit a placebo response. Not to mention homeopathy, violating several laws of physics for which dilution and shaking will allow a solution to retain the ‘memory’ of substances with which it has been in contact before [5]. A recent book [16] has exhaustively assessed the ethical aspects of CAM, exposing the different modes (moral, deontological, legal) currently violated by these health and wellness therapies. 

Statistical evaluation of results has become so important in clinical trials that its quality cannot be disconnected from ethical aspects. Thus, just as “medical research involving human subjects must conform to generally accepted scientific principles, as the Declaration of Helsinki states [17], it appears morally inescapable that also the inferential elaboration of data should square with the same high standard.

## Figures and Tables

**Table 1:** Classification scheme for the Bayes Factor, proposed by Jeffreys (1961).

Bayes Factor	Strength of Evidence
>100	Extreme evidence for H0
30–100	Very strong evidence for H0
10–30	Strong evidence for H0
3–10	Substantial evidence for H0
1–3	Anecdotal evidence for H0
1	No evidence
1/3–1	Anecdotal evidence for Ha
1/10–1/3	Substantial evidence for Ha
1/30–1/10	Strong evidence for Ha
1/100–1/30	Very strong evidence for Ha
<1/100	Extreme evidence for Ha
